# Exploration of Soil Functional Microbiomes—A Concept Proposal for Long-Term Fertilized Grasslands

**DOI:** 10.3390/plants11091253

**Published:** 2022-05-05

**Authors:** Vlad Stoian, Roxana Vidican, Păcurar Florin, Larisa Corcoz, Victoria Pop-Moldovan, Ioana Vaida, Sorin-Daniel Vâtcă, Valentina Ancuța Stoian, Anca Pleșa

**Affiliations:** 1 Department of Microbiology, Faculty of Agriculture, University of Agricultural Sciences and Veterinary Medicine Cluj-Napoca, Calea Mănăştur 3-5, 400372 Cluj-Napoca, Romania; vlad.stoian@usamvcluj.ro (V.S.); larisa.corcoz@usamvcluj.ro (L.C.); victoria.pop@usamvcluj.ro (V.P.-M.); 2 Department of Grasslands and Forage Crops, Faculty of Agriculture, University of Agricultural Sciences and Veterinary Medicine Cluj-Napoca, Calea Mănăştur 3-5, 400372 Cluj-Napoca, Romania; ioana.vaida@usamvcluj.ro (I.V.); anca.plesa@usamvcluj.ro (A.P.); 3 Department of Plant Physiology, Faculty of Agriculture, University of Agricultural Sciences and Veterinary Medicine Cluj-Napoca, Calea Mănăştur 3-5, 400372 Cluj-Napoca, Romania; sorin.vatca@usamvcluj.ro (S.-D.V.); valentina.stoian@usamvcluj.ro (V.A.S.)

**Keywords:** functional ecological niche, data analysis, dominance–codominance, functional guild, functional group

## Abstract

Exploring grassland microbiomes is a challenge in the current context of linking soil microorganism activity with the balance of these ecosystems. Microbiologists are constantly attempting to develop faster and lower-cost methods, and propose new and best-fitted indicators that will provide a more complex data analysis. A different concept was proposed for assessing functional microbiomes by splitting the functional ecological niche into complementary segments. The comparison with the upper and lower limits of the ecological niche provides a clearer image of community alterations due to long-term applied treatments. The method allows the extraction of the most sensitive and stable functional guilds, with the extraction of the most critical dominant–codominant functional groups in every segment of the functional niche. The resulting microbial functional–sociological model is ready to use on community-level physiological profile databases and also can be applied backward for vegetation analysis.

## 1. Introduction

Grasslands are dynamic ecosystems with high biological diversity and complex biogeochemical flows [1,2]. The interactions between the species overlapped on the pedo-climatic factor dynamics produce fluctuations and successions in natural vegetation in the dynamics of the pedo-climatic factors. The other way around, anthropization amplifies natural changes, resulting in a general reduction in ecosystem stability [3,4,5]. It creates the premise of reducing resilience, changing ecological niches over time, and decreasing the capacity to sustain a high diversity. An important element in grassland evolution can be observed by assessing the diversity of available microbiological functional substrates [6,7,8,9]. The specific competition of biological groups and additional resources stimulates the emergence of dominance–codominance phenomena supported by the interactions between species.

The study of phenomena and mechanisms that maintain the abundance of a species and the installation of dominance–codominance has a long scientific tradition [10]. Punctual evaluation with the metric frame (1 × 1 m), based on sub-replicated transects or with the help of the Braun-Blanquet scale allows obtaining excellent information on vegetation. At the same time, spatial–temporal analyses provide qualitative and quantitative indices to the evolution (or regression) of an ecosystem. All of these methods have procedure advantages such as how the speed of observation immediately results in extraction, combined with high sensitivity and specificity. Furthermore, α, β, and γ diversity could be analyzed, along with the equitability and phytocenoses climax assessment, together with grassland and plant association type [11,12,13]. Overall, the point where the species are defined was reached by the use of mathematical indicators, and also by dedicated software for complex vegetation analysis. The vegetation databases allow a realistic forecast of their future development, especially induced by the differentiated management.

However, all these advanced methodologies do not present maximum performance when applied to the biological counterpart of grasslands’ subsoil: the microflora [14]. Studies of grassland microbiology have evolved intensively in the last century [15,16,17,18,19]. In general, the evolution is restricted at the level of laboratory techniques. Furthermore, here, it causes a series of problems that restrict the development of microbiology as a component that could be easily integrated into grassland studies. The first issue is the highest percent of soil microflora that cannot be grown on culture media [20]. Microbial genomics had partially solved this issue: multiple genetic codes can already be identified, but they still do not possess a physical expression. Hence, large databases containing virtual taxa and taxonomic operational units have been created [21,22,23,24]. Unfortunately, they can only partially correlate with phytocoenosis species. A possible solution to this problem is the study of the microbial genetics of the rhizosphere. However, genetics is not entirely related to nutrient flows in this particular case, which leads to the need for a supplementary biochemical evaluation of the rhizosphere [25,26]. 

Due to expanding the sphere of microbiome understanding and the use of microbiology in grassland studies, the aim was set for a rapid method development to assess the functionality of soil microbial communities. The purpose of this article was to use Ecoplates to fully explore the changes in the functional profile of the microbial community in a natural mountain grassland fertilized over a long period. The tested hypotheses were formulated in the form of questions: (*a) Is the classical data analysis system recommended for CLPP (community-level physiological profile) by applying the ANOVA, PCA, and Diversity Indices tests sensitive enough to indicate the changes induced by differentiated fertilization?; (b) Can CLPP data undergo a further transformation before analysis to identify substrates where differences occur accurately?; (c) Does the data separation in differentiated matrices amplify the results of the ANOVA, PCA, and Diversity Indices tests?; (d) Could new created functional synthetic indices be able to explain the changes induced by fertilization?; (e) Could the concept of association in phytosociology be applied to identify the assembly patterns of functional microbial communities?; (f) Could a matrix model of Microbial Functional Sociology be built with high sensitivity for data, precise assessment, and which is adapted to CLPP-type tests?*

## 2. Results

### 2.1. The Conventional Approach

**Figure 1:**
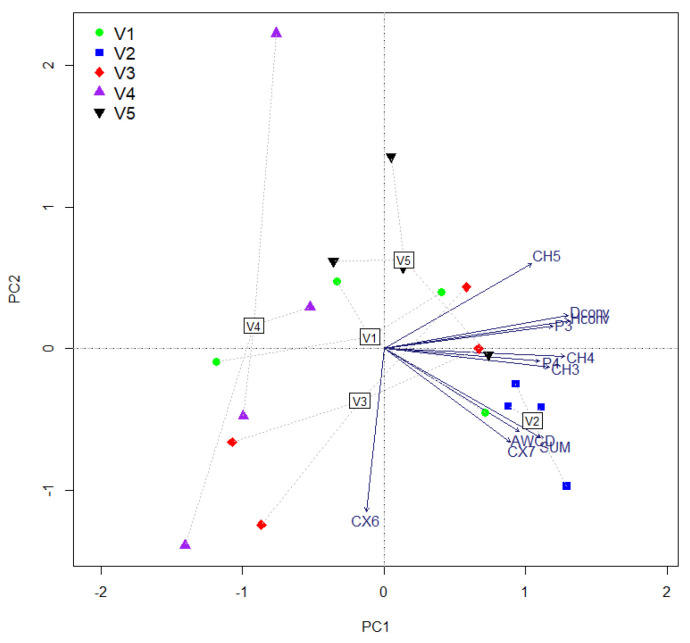
Simple PCA of microbial community physiological profile due to the application of treatments. (Variance explained by axes: Axis 1−28.68%, Axis 2−13.46%).

The conventional approach to CLPP revealed significantly reduced differences between microbial community parameters (Table 1). AWCD differs significantly only in the comparison between the treatment with 10 t ha^−1^ manure and the other treatments, especially compared with the mineral fertilized treatment. The diversity expressed by the Shannon and Simpson indices highlights significant differences only compared to the treatment treated with mineral fertilizers. PCA spatial exploration of microbial CLPP explains a total variance of only 42.14% and a number of 11 substrates in which a significant variation induced by manure treatments was observed (Figure 1). Both diversity indicators showed a strong change trend when low inputs were applied (V3), a tendency also followed by P3. Groups of P4, CH3, and CH4 are located in the same direction, with a deviation toward the unilateral application of organic fertilizer. This treatment was associated with the sum of activity and AWCD and CX7 as a significant group. Regardless of the fertilization applied, CH5 is presented as a group, being associated with both a non-fertilized and an organo-mineral-fertilized treatment (V3). One of the maximum fertilized treatments (V5) was fitted near this vector; V3 fertilization is beneficial to the stability of the CX6 group. The first observation of the whole experiment is that the stability of the treatments was reduced, even in the case of the control treatment. Decreased stability was associated both with the amount of microbial activity and with the group variations when applying the same treatment. The only treatment where a spatially restricted arrangement was observed is the one unilaterally fertilized with manure (V2).

### 2.2. Building the DEMSA Model

In response to the reduced suitability of the traditional methodology for the assessment of the results, a new model was developed for analyzing CLPP data. A Detailed Exploration of Microbial Sociological Assemblage (DEMSA) model was created to explore the complementary segments of the ecological niche occupied by the microbiome of the HNV (high natural value) grassland fertilized for a long period. The substrates on the Biolog EcoPlates were chemically separated into functional classes (functional guild), and each substrate is considered a functional group (Supplementary Material). The concept and formulas used in this approach were developed based on the theoretical concept formulated by Vidican et al. [27] to evaluate the exchange pressure in soil microbiomes due to fertilizers and pesticides. The changes made to the calculation formulas allowed the complete exploration of the functional soil microbiome and the development of the excel used in assembling the model (Supplementary Table S1). All the formulas used in the DEMSA model approach were designed to make visible any change or difference registered in functional microbiomes. The development of stand-alone matrices, as a result of comparison with the control variation range, allows establishing the size of the soil’s native functional ecological niche. Further data analysis by comparing the use level of the substrates with the average and the limits of the control interval allows the highlighting of changes due to fertilization over a long period and the elimination of the masked effects.

**Figure 2:**
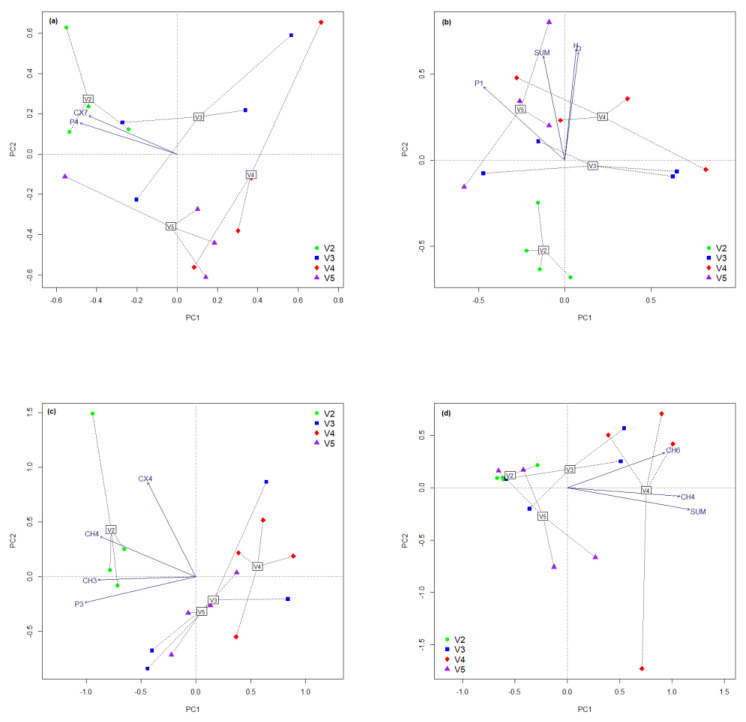
PCA of complementary segments of ecological niche associated with microbial community physiological profile due to the application of treatments. Variance explained by axis for each PCA in brackets. (**a**) Intensification (Axis 1−20.93%, Axis 2−16.49%); (**b**) Expansion (Axis 1−35.94%, Axis 2−14.24%); (**c**) Narrowing (Axis 1−22.60%, Axis 2−17.20%); (**d**) Contraction (Axis 1−27.47%, Axis 2−25.48%).

The exploration of complementary segments brings a higher analysis sensitivity for grassland soil microbiomes. The application of the standard data analysis methodology to the new data matrix sets provides a clarification of the variations in image regarding the microbial groups from the ecological niche (Figure 2a–d, Table 2). The amount of microbial activity in the intensification segment highlights a significantly lower increase in activity, especially at high fertilization values. The reduction trend is also observed at the level of the Shannon index; in the case of V4 and V5 the values approach an average level of activity. The total variance expressed by PCA for this segment is close to the conventional analysis, but much more balanced between axes. The powerful (expansion) responding microbial groups are CX7 and P4, associated with the lowest fertilization. The positioning of the treatment on the ordination indicates spontaneous and elastic reactions of the microbial groups, regardless of the applied treatment. Further exploration in the area of expansion of the ecological niche highlights the beneficial effect of reduced fertilization (V2). Treatments with high amounts of mineral fertilizers (V4 and V5) lead to a significant reduction in microbial expansion compared to V3. In this segment, there is a slight restriction of the microbial functional community heterogeneity, supported by the average effect of the treatments. The reaction to the treatments is visible on the PCA at the level of the functional groups CX4, CH4, CH3 and P3, all having the gradient oriented opposite to the mineral fertilizers. There is an increase in the stability of the treatments, which supports the similar associations of microbial groups. Exploration of the narrowing and contraction segments of the ecological niche highlights a 2% higher variance in both PCAs (Table 2). The amount of reduced microbial activity was less than 2 for the fully organic treatment (V2). The application of a mineral fertilizer leads to a significant gradual restriction of microbial activity, with maximum values for integral mineral fertilization. The narrowing observed in the microbial communities is homogeneous (Simpson index in the range 0.7–1.0), with significant differences between the experimental treatments. The only microbial group considered extremely sensitive to fertilization in this segment is P1, oriented towards organo-mineral fertilization with high values (V5). The amount of activity that can be restricted within the ecological niche is due to the increase in the level of treatments, while the effect of fertilization and community homogeneity is related only to mineral fertilization (V4). The contraction of the ecological niche is very visible at the integral mineral treatment (V4), with similar contractions in the case of organo-mineral fertilization (V3 and V5). Shannon diversity indicates a strong point effect on a small number of functional groups in the case of organic fertilization; the group diversity was also increased by the addition of mineral treatments. The Simpson index supports the grouped reaction in the case of reduced treatments, with insignificant differences in the case of the mineral fertilizer addition. The PCA analysis of contraction highlights only two functional groups (CH6 and CH4) were extremely sensitive to the application of mineral fertilizers. The total microbial activity amount that can be contracted was positioned in opposition to organic fertilizers. All treatments with organic fertilizers reacted in groups to the input pressure, and the distances from the center of each group were reduced. Determining the size of the ecological niche based on the microbial group activity patterns, with maximum and minimum limits, allows the separation of fluctuations induced by treatments into four complementary segments. The intensification evaluates the observed increases in the microbial groups placed in the middle–maximum limit range of the ecological niche. Above the maximum limit, the increases are considered an expansion of the ecological niche. Conversely, the reduction in microbial activity in the medium–minimum range restricts a part of the microbial community within the normal limits of the ecological niche, and anything that exceeds this level is considered a contraction of the ecological niche associated with management.

The application of inputs acts specifically on the functional communities in the grasslands, modifying the metabolic patterns at the level of each segment of the ecological niche (Table 2). The observed intensification of the ecological niche indicates significantly higher values of the CH guild associated with the integral organic fertilization (V2), respectively, equal to the AA and CX groups associated with the reduced organo-mineral fertilization (V3). The intensification index of microbial communities reaches a maximum of 7.23–7.26% in variants V2 and V3, decreasing in variants with high treatments. The association of guilds represents combinations of CH, CX, and AA, identical in the case of V2 and V5 and with an interchange in the case of V3 and V4. The dominance of the main microbial groups is different at each fertilization, with the presence of group CX6 in the organically treated variant (V2) and the presence of AA2 (V3), AA3 (V4), respectively, and AA4 (V5), with all these dominants belonging to the same AA group. Restrictions considered within the boundaries of the ecological niche indicate higher overall values as the treatment gradient. The restriction index indicates significant differences of 2% between the organic whole treatment (V2) and the one to which low mineral inputs are applied (V3), respectively, a maximum of 8.74% when applying all mineral inputs. The association of guilds presents the sensitivity of only two guilds (CH and CX), but with diversified dominance of functional groups. The only treatment that leads to the dominant reduction in a group from another guild is high organo-mineral fertilization (V5). The analysis of the expansion potential highlights the same dominant guild (CH) in the case of treatments containing manure and CX in the integral mineral treatment. Dominance follows the same trend, with the presence of P3 associated with manure and CX6 at mineral inputs. In contrast, the expansion index shows a high potential for V2 and V3 (>10%) and a reduction in this potential to below 7% when adding high doses of mineral inputs (V4 and V5). In terms of contraction of the ecological niche, the additional application of mineral inputs leads to high losses, but without discrimination between guilds. The dynamics are interesting of dominant contraction of CH9 in the variants with unilateral inputs (V2 and V4), respectively, these belonging to the same guild as the dominant ones from the organo-mineral treatments (V3 and V5). The contraction index reaches a maximum of 16% at mineral inputs (V4), and is maintained in the range of 6–8% by applying organo-mineral treatments (V3 and V5).

The functional alteration of the microbial community (Table 2) acts as an indicator of the promoted change of fertilization, in the case of the application of organic fertilizers (V2) being found to induce an oversizing of the functional community (18%). When applying mineral fertilizers, a reduction in the microbial community is observed by more than 10% (V4). The difference of only 50 kg ha^−1^ N and 25 kg ha^−1^ PK between V3 and V5 leads to two different directions of quantitative evolution of microbial communities. In the case of V3, a slight increase is observed, while with the additional application of mineral fertilizers, the microbial community tends to decrease. Changes in the microbial functional profile are visible both within the communities on which identical treatments were applied and in the comparison between communities. Within the microbial communities, the most stable treatment variant is V3 (10 t ha^−1^ manure + 50N25PK), with a similarity of the communities of 89.65%, with significant differences compared to the other treatments. This aspect indicates the potential for different evolutionary directions, with clear separations due to fertilization gradients. 

The highest value of similarity between communities is at V4; mineral fertilization reduces the size of the community and a total of 86% of the community is present in the other variants (Table 2). There are trends in the change in community compared to that supported by the unilateral application of manure, but with differences given by the additional doses of nitrogen applied (V3 and V5). The lowest share of the community in the other variants is when applying unilateral organic fertilization (V2), with a percentage of only 75% present in the other communities. 

The intensifications and narrowings of the microbial functional communities remain within limited limits, with a strong opposition between the manure-treated and the mineral-treated version (Table 2). On the other hand, with the exploration of the expansion and contraction segments, a greater distance between communities is observed. Expansion is associated with two carbohydrates (CH3 and CH4), respectively, P3, CX4, and the sum of microbial activity. The greatest expansion is observed in the treatment with manure, in opposition to the treatments with mineral fertilizers that restrict the expansive functional diversity. The contraction of the microbiome is stronger in the case of mineral treatments, but also with a stable dispersion of the contraction potential. The association of the gradients of the functional groups with this segment of the ecological niche is defined by three carbohydrates (CH6, CH8, and CH9), respectively, AA6 and AM1. The contraction has a lower lateral deviation compared to the expansion of the microbiome, which supports a grouped response in case of reduced microbial activity. In the opposite direction, microbial diversity supports the succession and alternation of functional groups with the same activity in the decomposition of specific substrates.

Functional group associations showed different patterns when applied to each complementary segment of the niche (Table 2), which overall sustain the appearance of distinct guild associations. This information can be further used for a better understanding of the soil microbiome’s functionality, with the elimination of the masked effects. As a response to phytosociology, microbial functional sociology applied to Complementary segments in the ecological niche provide multiple specific guild associations (completed by functional groups’ dominance–codominance). The intensification sustains as the first guild CH (organic) and AA (mineral), both with CX as the second guild. Except for mineral treatment, for all organic related treatments, CH is the main guild in case of expansion. Narrowing and contraction are also related to CX and CH, but overall, there is a change in functional group dominance–codominance.

**Figure 3:**
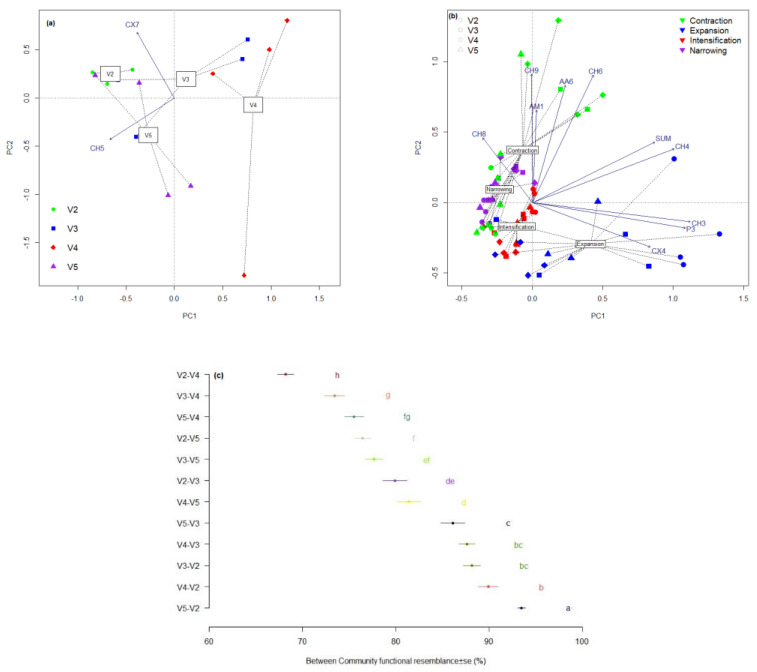
PCA profile of (**a**) sharing in the microbial community (variance explained by axis: Axis 1−26.49%, Axis 2−22.14%) and (**b**) complementary segments of ecological niche associated with microbial community physiological profile (variance explained by axis: Axis 1−24.79%, Axis 2−13.31%) and (**c**) LSD comparison of community overlaps due to the application of treatments (different letters near to average ± s.e. present significant differences at *p* < 0.05). V1−Control; V2−10 t ha^−1^ manure; V3−10 t ha^−1^ manure + N50, P_2_O_5_ 25 kg ha^−1^, K_2_O 25 kg ha^−1^; V4−N100, P_2_O_5_ 50 kg ha^−1^ K_2_O 50 kg ha^−1^; V5−10 t ha^−1^ manure + N100, P_2_O_5_ 50 kg ha^−1^ K_2_O 50 kg ha^−1^.

Graphically, the positioning in the same area of the variants treated with manure is visible compared to the mineral fertilized one (Figure 3a–c). The location of the PCA of the size of the common microbiome with that of the control indicates stability and a reduced variation in the variant fertilized only with manure (Figure 3a). This community is bordered by the gradients of substrates CX7 and CH5, each of these substrates having a high potential for modification associated with a specific treatment. In the case of CX7, the combination is with manure treated with reduced doses of mineral fertilizers (V3), while increasing the level of the dose of mineral fertilizers changes the association to CH5. The total variance explained by PCA is 48.63% and highlights the strong disruptive potential of mineral fertilization (V4), with the location of the samples being very distant. Organo-mineral fertilization is responsible for the successions in the functional microbiome; both variants with this type of treatment (V3 and V5) have components close to V2 (manure) and V4 (mineral fertilization). The use of a segmentation model of the ecological niche brings multiple advantages (Figure 3b). The total variance in the PCA is 38.10% with a graphical exposure of a high number of gradients of the functional groups. The four complementary segments of the ecological niche follow one another in a semicircular pattern, with the limits represented by the expansion and contraction segments.

The presence of identical functional groups in the communities treated differently (Figure 3c) highlights the opposite poles occupied by the organic treatment compared to the mineral one. In the case of organic treatment (V2), the increase in total microbial activity causes the activity found in the other treatments to be 90% present in this community. The differences are small between the overlap of communities up to about 85%; below this value there are significant gradual differences. The application of mineral fertilizers in addition to manure (V3 and V5) is sufficient to support a similarity of the community in the range of 70–80% with the community defined by the integral mineral treatment (V4). In contrast, the microbial community defined by organic fertilization (V2) is present in a percentage below 70% in that defined by mineral treatment (V4).

## 3. Discussion

### 3.1. Previous Approaches and the Importance of CLPP Analysis in Grasslands

Grassland ecosystems are characterized by high diversity and are the main key to maintaining flora, fauna, and human populations around the world [28]. High plant biodiversity is often reflected in the diversity of fauna and microorganisms [29]. Between these three categories, different specific interrelationships give uniqueness to this ecosystem. High soil biodiversity reflects a set of functional roles, and the soil microbial community is divided into three functional groups [30]. Around 34% of the global terrestrial carbon is stored in grasslands, being quite fragile in terms of stability [31]. The soil of these ecosystems has the highest carbon content of most terrestrial ecosystems [32], about three times more carbon than in flora and twice as much as in the atmosphere [33]. From an ecological point of view, the soil is the substrate responsible for increasing plant productivity, maintaining water and air quality, and supporting a diverse community of microorganisms [34]. Soil microbial biomass intervenes in 90% of ecosystem functions so it is essential for establishing soil quality. Microbial communities are important for providing vital services to the ecosystem, by supplying plants with water and nutrients, conserving soil structure, and distributing water [35]. Grassland ecosystems are key components of ecological communities on Earth [36] and provide ecological services such as carbon sequestration, climate regulation, and biodiversity maintenance [37]. These ecosystems are also involved in the water and nutrient cycles through which soil functions are maintained [38]. The grassland’s soil is a deposit of microorganisms of immense diversity. These are important for climate change mitigation, immobilization, and sequestration of greenhouse gases [39]; additionally, microorganisms are vital to support soil fertility for the entire ecosystem, regulation of nutrients, along with the distribution and decomposition of organic matter [40].

Bacteria and fungi are part of chemical regulators because they break down organic matter and contribute to the flow of nutrients. Depending on the conditions of land management or use and the abiotic and biotic processes present in the soil, the microbial communities in the grassland soil can change radically [40]. These changes occur in the activity and composition of microbial communities in grassland soils. Changes in the assemblage of grassland plant groups are very visible in the diversification and the composition of microbial associations [41]. The basic property of the soil in mountain grasslands, its fertility, is correlated with the value of biomass represented by microbial communities. High biomass of microbial communities is found in soils with a low fertility content and in situations of high fertility the microbial biomass is decreasing. This creates the premises, in last few decades, for a major interest toward world sustainability as ecosystem degradation becomes increasingly evident.

Various tests are used to quantify and determine the dimension and functions of soil microbial communities. Therefore, knowledge of soil microbiota functions is important to properly manage the processes that control nutrient availability to plants [42]. Different methods and tests are used to quantify the effects caused by large or small amounts of inputs: nitrogen, phosphorus, potassium, carbon, and heavy metals. The study of microbial biodiversity could be performed with the development of biotechnologies, by using microbial DNA, and can obtain accurate information about the diversity of microorganisms. As a rapid alternative, numerous researchers have observed that the method of substrate decomposition, based on Biolog EcoPlates, is a viable and effective method to assess the microbial functionality in the ecosystem [43]. The Biolog EcoPlates method is used to present physiological profiles at a community level and is relatively easy to use, with cheaper techniques and provides faster results. Depending on the biochemical and biological properties, this method can determine the ecological status of soil and water samples [44]. CLPP is considered to be land use-, soil management-, and soil-texture-specific and reflects some aspects of functional diversity rather than community structure [45]. Microbial communities are related to the succession gradient of vegetation, in terms of functional structure [46,47], and the large database of results offers a support in the complex analysis of soil microbial–plant communities.

### 3.2. What They Have: Specificity That Brings Performance in the Vegetation Analysis

Phytosociology offers tools with high specificity for the vegetation’s rapid and realistic assessment [48]. HNV grasslands require both assessments of the phytocoenosis stability and characteristics and for the specific assemblage of the component species interactions. The stability of phytocenoses is dependent on the structural and functional complexity, so the greater the diversity is, the greater the multitude of evolutionary possibilities that could be identified [49]. The use of coeno-ecological groups reflects the species requirements towards ecological factors and the coenotic behavior is visible in the frequencies of occurrence, coverage, vitality, and competitiveness. This approach allows a biological assembly and stratification that indicates the stage of a plant unit. In addition, the grasslands are evaluated from agronomic perspectives, which present an assembly of species in functional groups: grasses, legumes, other botanical families [50]. The system of agronomic classification of grasslands uses grasses as a defining group and the name of the grassland type is usually the association of the first two grass species. It is an assessment proposal that allows the visualization of fluctuations and temporal successions of the plant community, which can later be supplemented with the ecological requirements of each species (e.g., Ellenberg—[51]). The agro-ecological spectrum resulting from this type of assessment defines the species pattern present in this type of phytocoenosis.

### 3.3. The Most Frustrating Questions: What Don’t Microbiologists Have?

Enzymology, chemistry, biochemistry, and the analysis of the reaction induced by a certain type of substrate manage to increase the level of understanding of the microbial communities’ general functioning [52,53]. Additionally, at this point, there are three major questions (problems) about the results obtained. **The first** is given by the complex cost–time–personnel–technology component of studies on microbial diversity in grasslands. While the analysis of vegetation involves a low cost and number of people, the results are obtained immediately after the completion of field observations. For the analysis of microbiomes, additional training is required. There are costs related to the sample collection and preservation for laboratory analysis. The analysis technique is expensive, and the staff is very specialized [54,55]. **The second** is data analysis. For vegetation, there are a number of traditional, widely used methods and numerous models. Vegetation can be interpreted historically, up to the maximum simplification of the presence/absence type. In addition, vegetation study methods have standardized and verified indicators over a long period of time. This is not the case with microbiome analysis. In general, different vegetation indicators and methodologies have been adapted to microbiological analyses, but without perfect folding [56]. **The third** problem is given by the agronomic and ecological relevance. The high costs of microbiological analyzes severely restrict the number of sites/samples that can be analyzed. Over time, disparate and staggered results have been obtained that do not allow the interpolation and identification of the real value of biotic and abiotic pressures in grasslands. Therefore, it highlights the need for identification fast and with lower-cost methods that can be performed simultaneously (or at most in a short period) compared to vegetation analysis. This type of study is needed to create new standards and integrate microbiome analysis into current analyses of a grassland ecosystem.

### 3.4. In Search of Higher Sensitivity in Data Analysis of Microbial Functional Communities

The analysis of microbial communities in grasslands is a paradox: the innumerable results were obtained through a high number of methodologies and techniques, but with a reduced capacity for harmonization and an even lower capacity for interpretation. CLPP analyses have the advantage of speed and provide clues on how to use the substrates. A second advantage is the use of the same standard (31 + 1 substrates) applied as a pattern in different phytocenoses, which allows comparisons between ecosystems populated by different species and diversity. Still, subject to the methods of analysis typical for vegetation (presence/absence; % of the community; indices of diversity; spatial ordination), this type of result is not able to reveal subtle changes in microbiomes. An additional point in the vegetation analysis is the presence or the absence of a species, an aspect that is not highlighted by CLPP due to the microbial diversity capable of decomposing the same substrate. In this context, even if the data analysis can be performed with vegetation methods, segmentation of the functional microbiology data and the development of new indicators are necessary.

### 3.5. The DEMSA Concept—Linkage to Past, Present, and Future Studies

As Ramette [14] postulated, most of the studies in the field of microbial ecology use vegetation procedures for data analysis, which, for microbial communities, represents low performance and the possibility of losing/avoiding the best exploration. Additionally, the current trend in soil microbial studies is to identify procedures with lower costs, higher resolution and with results delivered fast. Soil microbiomes can be better explored by the DEMSA model in separate segments of the functional ecological niche. The model is fully adapted to re-analyze the previous data produced in Ecoplates studies, with a better exploration of small/masked differences. For present and future studies, the DEMSA can be applied both for soil microbiomes and vegetation analysis. The concept is based on functional units—each substrate represents a functional group and multiple similar substrates form a functional guild. Microbial functional sociology is similar to phytosociology, and therefore permits an in-depth exploration and analysis, separately for the observed communities’ increases and decreases. The DEMSA approach will improve the correlation between vegetation and microbiome analysis. An important point is how steps forward were made, along with the proposal of a new class of indicators with in-depth overview resolution on microbiomes.

## 4. Materials and Methods

Soil samples were collected in 2020, from a long-term experiment established in 2001 in a natural grassland in the Apuseni Mountains, Romania [50,57]. The field experiment followed the reaction and changes in the plant community assemblage due to the application of mineral, organic, and organo-mineral fertilizers. All 5 treatments, in 4 replications, were represented by the inputs’ gradient and type of fertilizer: V1—control, non-fertilized; V2—10 t ha^−1^ manure; V3—10 t ha^−1^ manure + N_5_0, P_2_O_5_ 25 kg ha^−1^, K_2_O 25 kg ha^−1^; V4—N_100_, P_2_O_5_ 50 kg ha^−1^ K_2_O 50 kg ha^−1^; V5—10 t ha^−1^ manure + N_100_, P_2_O_5_ 50 kg ha^−1^ K_2_O 50 kg ha^−1^.

For Ecoplates tests, soil and procedures followed the method described by [58], with all substrates being organized based on their chemical similarity (Table 3). The solution inoculated in Ecoplates was diluted up to 10^−4^, and the reading was conducted at 590 nm with a plate reader. The entire process lasted 5 days, up to the plateau phase, when no increases were observed in the readings.

Data analysis followed primarily the classic procedures [59]; for all data the AWCD (average well color development) and the diversity indices Shannon and Simpson were calculated. Additionally, the Principal Component Analysis (PCA) was conducted for a better results exploration. All data were analyzed with R Studio Software [60] within the R platform [61], with “vegan” [62] and “agricolae” [63] packages. The new method for data analysis in this article followed the concept proposed by [27], with all the synthetic indicators created to visualize even the smallest differences between functional microbiomes.

## Figures and Tables

**Table 1:** Functional microbiome dimension and indicators due to applied treatments.

	Sum	AWCD	H	D
V1	50.7 ± 3.54 bc	1.35 ± 0.09 bc	3.31 ± 0.03 a	0.96 ± 0.00 a
V2	59.9 ± 0.71 a	1.65 ± 0.03 a	3.37 ± 0.00 a	0.96 ± 0.00 a
V3	54.2 ± 0.91 b	1.45 ± 0.04 b	3.31 ± 0.03 a	0.96 ± 0.00 a
V4	45.4 ± 1.03 c	1.25 ± 0.03 c	3.23 ± 0.02 b	0.95 ± 0.00 b
V5	48.9 ± 0.93 bc	1.35 ± 0.03 bc	3.34 ± 0.01 a	0.96 ± 0.00 a
F test	9.55	7.27	4.40	4.90
*p* value	*p* < 0.001	0.002	0.015	0.010

Note: Means ± s.e. followed by different letters indicates significant differences at *p* < 0.05. V1−Control; V2−10 t ha^−1^ manure; V3−10 t ha^−1^ manure + N50, P_2_O_5_ 25 kg ha^−1^, K_2_O 25 kg ha^−1^; V4−N100, P_2_O_5_ 50 kg ha^−1^ K_2_O 50 kg ha^−1^; V5−10 t ha^−1^ manure + N100, P_2_O_5_ 50 kg ha^−1^ K_2_O 50 kg ha^−1^.

**Table 2:** Changes in Functional Microbiome, Guild Association, and Group Dominance–Codominance due to treatments.

Matrix of	Intensification	Narrowing	Expansion	Contraction
AA				
V2	0.98 ± 0.05 ab	0.15 ± 0.09 c	0.77 ± 0.23 a	0.01 ± 0.01 a
V3	1.23 ± 0.15 a	0.24 ± 0.09 bc	0.91 ± 0.24 a	0.55 ± 0.31 a
V4	1.01 ± 0.06 ab	0.49 ± 0.06 ab	0.79 ± 0.26 a	0.66 ± 0.24 a
V5	0.65 ± 0.15 b	0.71 ± 0.12 a	0.38 ± 0.30 a	0.57 ± 0.12 a
F test	4.07	6.37	0.77	1.96
*p* value	*0.033*	*0.008*	*0.534*	*0.174*
AM				
V2	0.18 ± 0.07 a	0.22 ± 0.11 a	0.10 ± 0.07 a	0.00 ± 0.00 b
V3	0.08 ± 0.03 a	0.25 ± 0.06 a	0.11 ± 0.11 a	0.17 ± 0.12 b
V4	0.12 ± 0.08 a	0.37 ± 0.07 a	0.07 ± 0.05 a	0.92 ± 0.21 a
V5	0.09 ± 0.05 a	0.24 ± 0.09 a	0.10 ± 0.07 a	0.26 ± 0.19 b
F test	0.51	0.55	0.05	6.47
*p* value	*0.681*	*0.657*	*0.982*	*0.007*
CH				
V2	1.23 ± 0.06 a	0.63 ± 0.09 c	2.83 ± 0.32 a	0.48 ± 0.20 b
V3	0.94 ± 0.16 ab	1.02 ± 0.14 b	1.90 ± 0.21 b	1.64 ± 0.59 ab
V4	0.60 ± 0.10 b	1.36 ± 0.09 a	0.46 ± 0.15 c	2.98 ± 0.50 a
V5	0.91 ± 0.15 ab	0.81 ± 0.06 bc	1.38 ± 0.24 b	0.84 ± 0.54 b
F test	3.86	8.98	16.54	5.22
*p* value	*0.038*	*0.002*	*p* < *0.001*	*0.015*
CX				
V2	1.11 ± 0.09 ab	0.73 ± 0.17 b	1.65 ± 0.16 a	0.27 ± 0.22 b
V3	1.23 ± 0.18 a	0.87 ± 0.17 ab	1.65 ± 0.38 a	0.40 ± 0.13 b
V4	0.82 ± 0.14 ab	1.17 ± 0.17 ab	1.61 ± 0.40 a	2.02 ± 0.75 a
V5	0.65 ± 0.18 b	1.27 ± 0.15 a	0.63 ± 0.20 b	1.65 ± 0.44 ab
F test	2.75	2.26	2.63	3.71
*p* value	*0.089*	*0.134*	*0.098*	*0.043*
P				
V2	0.81 ± 0.09 a	0.08 ± 0.07 b	2.11 ± 0.30 a	0.02 ± 0.02 a
V3	0.44 ± 0.12 b	0.41 ± 0.06 a	1.05 ± 0.41 b	0.46 ± 0.30 a
V4	0.23 ± 0.08 bc	0.54 ± 0.16 a	0.17 ± 0.12 b	0.67 ± 0.31 a
V5	0.14 ± 0.07 c	0.57 ± 0.05 a	0.62 ± 0.22 b	0.41 ± 0.17 a
F test	9.61	5.00	8.28	1.29
*p* value	*0.002*	*0.018*	*0.003*	*0.322*
Index of	Intensification	Narrowing	Expansion	Contraction
V2	7.23 ± 0.19 a	3.07 ± 0.16 c	12.4 ± 0.73 a	1.36 ± 0.44 c
V3	7.26 ± 0.72 a	5.20 ± 0.56 b	10.4 ± 0.83 a	6.06 ± 1.95 b
V4	6.17 ± 0.41 ab	8.74 ± 0.75 a	6.79 ± 1.24 b	16.0 ± 1.41 a
V5	5.03 ± 0.43 b	7.42 ± 0.47 a	6.39 ± 0.33 b	7.78 ± 1.79 b
F test	4.76	21.83	11.83	16.25
*p* value	*0.021*	*0.000*	*0.001*	*0.000*
Guild Association				
V2	CH–CX	CX–CH	CH–P	CH–CX
V3	CX–AA	CH–CX	CH–CX	CH–AA
V4	AA–CX	CH–CX	CX–AA	CH–CX
V5	CH–CX	CX–CH	CH–CX	CX–CH
Group Association Dominance–Codominance	Intensification	Narrowing	Expansion	Contraction
V2	CX6–CH2	CH7–CX3	P3–CX4	CH9–CH1
V3	AA2–CX6	CX7–AM1	P3–CH3	CX1–CH6
V4	AA3–AA2	CX7–AM1	CX6–CX4	CH9–AM1
V5	AA4–AA3	AA1–CX1	P3–CH3	CX7–AA1
Community comparison	Functional alteration	Within-community functional resemblance	Between-community functional resemblance	
V2	18.07 ± 1.41 a	80.64 ± 1.78 b	74.83 ± 0.92 c	
V3	6.92 ± 1.81 b	89.65 ± 0.40 a	79.75 ± 1.06 b	
V4	−10.44 ± 2.04 d	81.36 ± 1.14 b	86.32 ± 0.80 a	
V5	−3.53 ± 1.85 c	77.38 ± 1.85 b	85.04 ± 1.21 a	
F test	48.40	13.59	27.35	
*p* value	*p* < *0.001*	*p* < *0.001*	*p* < *0.001*	

Note: Means ± s.e. followed by different letters indicates significant differences at *p* < 0.05. CH—Carbohydrates; P—Polymers; CX—Carboxylic and acetic acids; AA—Amino acids; AM—Amines/amides. Full name of substrates is provided in Section 4.

**Table 3:** Functional groups and guilds based on substrates in Biolog Ecoplates.

Substrate	Code	Substrate	Code
Water	W	d-Galactonic acid γ-lactone	CX2
Pyruvic acid methyl ester	CH1	d-Galacturonic acid	CX3
Tween 40	P1	2-Hydroxy benzoic acid	CX4
Tween 80	P2	4-Hydroxy benzoic acid	CX5
α-Cyclodextrin	P3	γ-Hydroxy butyric acid	CX6
Glycogen	P4	Itaconic acid	CX7
d-Cellobiose	CH2	α-Keto butyric acid	CX8
α-d-Lactose	CH3	d-Malic acid	CX9
β-Methyl-d-glucoside	CH4	l-Arginine	AA1
d-Xylose	CH5	l-Asparagine	AA2
i-Erythritol	CH6	l-Phenylalanine	AA3
d-Mannitol	CH7	l-Serine	AA4
N-Acetyl-d-glucosamine	CH8	l-Threonine	AA5
d-Glucosaminic acid	CX1	Glycyl-l-glutamic acid	AA6
Glucose-1-phosphate	CH9	Phenylethylamine	AM1
d,l-α-Glycerol phosphate	CH10	Putrescine	AM2

Note: CH—Carbohydrates; P—Polymers; CX—Carboxylic and acetic acids; AA—Amino acids; AM—Amines/amides, based on [58] classification.

## Data Availability

The data presented in this study are available on request from the corresponding authors.

## Supplementary Materials

The following supporting information can be downloaded at: www.mdpi.com/article/10.3390/plants11091253/s1, Supplementary data: Full description of the DEMSA model; Table S1: DEMSA calculation excel.

## References

[1] Duru M., Pontes L.D.A.S., Schellberg J. (2019). Grassland Functional Diversity and Management for Enhancing Ecosystem Services and Reducing Environmental Impacts.

[2] Rumpel C., Crème A., Ngo P.T., Velásquez G., Mora M.L., Chabbi A. (2015). The Impact of Grassland Management on Biogeochemical Cycles Involving Carbon, Nitrogen and Phosphorus. J. Soil Sci. Plant Nutr..

[3] Aubree F., David P., Jarne P., Loreau M., Mouquet N., Calcagno V. (2020). How Community Adaptation Affects Biodiversity–Ecosystem Functioning Relationships. Ecol. Lett..

[4] Naeem S., Duffy J.E., Zavaleta E. (2012). The Functions of Biological Diversity in an Age of Extinction. Science.

[5] Vogel A., Scherer-Lorenzen M., Weigelt A. (2012). Grassland Resistance and Resilience after Drought Depends on Management Intensity and Species Richness. PLoS ONE.

[6] Michalska-Smith M., Song Z., Spawn-Lee S.A., Hansen Z.A., Johnson M., May G., Borer E.T., Seabloom E.W., Kinkel L.L. (2021). Network Structure of Resource Use and Niche Overlap within the Endophytic Microbiome. ISME J..

[7] Sasse J., Martinoia E., Northen T. (2018). Feed Your Friends: Do Plant Exudates Shape the Root Microbiome?. Trends Plant Sci..

[8] Wang G., Schultz P., Tipton A. (2019). Soil Microbiome Mediates Positive Plant Diversity-Productivity Relationships in Late Successional Grassland Species. Ecol. Lett..

[9] Wu S.-H., Huang B.-H., Huang C.-L., Li G., Liao P.-C. (2018). The Aboveground Vegetation Type and Underground Soil Property Mediate the Divergence of Soil Microbiomes and the Biological Interactions. Microb. Ecol..

[10] Van der Maarel E., Franklin J. (2012). Vegetation Ecology.

[11] Janišová M., Michalcová D., Bacaro G., Ghisla A. (2014). Landscape Effects on Diversity of Semi-Natural Grasslands. Agric. Ecosyst. Environ..

[12] Meier E.S., Hofer G. (2016). Effects of Plot Size and Their Spatial Arrangement on Estimates of Alpha, Beta and Gamma Diversity of Plants in Alpine Grassland. Alp Bot..

[13] Gibson D.J. (2009). Grasses and Grassland Ecology.

[14] Ramette A. (2007). Multivariate Analyses in Microbial Ecology. FEMS Microbiol. Ecol..

[15] Brodie E., Edwards S., Clipson N. (2003). Soil Fungal Community Structure in a Temperate Upland Grassland Soil. FEMS Microbiol. Ecol..

[16] Diamond S., Andeer P.F., Li Z., Crits-Christoph A., Burstein D., Anantharaman K., Lane K.R., Thomas B.C., Pan C., Northen T.R. (2019). Mediterranean Grassland Soil C–N Compound Turnover Is Dependent on Rainfall and Depth, and Is Mediated by Genomically Divergent Microorganisms. Nat. Microbiol..

[17] Felske A., Wolterink A., Van Lis R., De Vos W.M., Akkermans A.D.L. (2000). Response of a Soil Bacterial Community to Grassland Succession as Monitored by 16S RRNA Levels of the Predominant Ribotypes. Appl. Environ. Microbiol..

[18] Griffiths R.I., Whiteley A.S., O’Donnell A.G., Bailey M.J. (2003). Influence of Depth and Sampling Time on Bacterial Community Structure in an Upland Grassland Soil. FEMS Microbiol. Ecol..

[19] Navrátilová D., Tláskalová P., Kohout P., Dřevojan P., Fajmon K., Chytrý M., Baldrian P. (2019). Diversity of Fungi and Bacteria in Species-Rich Grasslands Increases with Plant Diversity in Shoots but Not in Roots and Soil. FEMS Microbiol. Ecol..

[20] Bodor A., Bounedjoum N., Vincze G.E. (2020). Challenges of unculturable bacteria: Environmental perspectives. Rev. Environ. Sci. Biotechnol..

[21] Delgado-Baquerizo M. (2019). Obscure Soil Microbes and Where to Find Them. ISME J..

[22] Choi J., Yang F., Stepanauskas R. (2017). Strategies to improve reference databases for soil microbiomes. ISME J..

[23] Wang C., Tang Y. (2019). A Global Meta-Analyses of the Response of Multi-Taxa Diversity to Grazing Intensity in Grasslands. Environ. Res. Lett..

[24] Zhou F., Ding J., Li T., Zhang X. (2020). Plant Communities Are More Sensitive than Soil Microbial Communities to Multiple Environmental Changes in the Eurasian Steppe. Glob. Ecol. Conserv..

[25] Grayston S.J., Griffith G.S., Mawdsley J.L. (2001). Accounting for Variability in Soil Microbial Communities of Temperate Upland Grassland Ecosystems. Soil Biol. Biochem..

[26] Ritz K., McNicol J.W., Nunan N. (2004). Spatial Structure in Soil Chemical and Microbiological Properties in an Upland Grassland. FEMS Microbiol. Ecol..

[27] Vidican R., Stoian V., Șandor M., Ozunu A., Nistor I.A., Petrescu D.C. (2017). Fertilization and Pesticides as Elements of Pressure on Microbial Communities.

[28] Dengler J., Janišová M., Török P., Wellstein C. (2014). Biodiversity of Palaearctic Grasslands: A Synthesis. Agric. Ecosyst. Environ..

[29] De Deyn G.B., Raaijmakers C.E., Zoomer H.R., Berg M.P., de Ruiter P.C., Verhoef H.A., Bezemer T.M., Putten W.H. (2003). van der Soil Invertebrate Fauna Enhances Grassland Succession and Diversity. Nature.

[30] Saccá M.L., Barra Caracciolo A., Di Lenola M., Grenni P., Lukac M., Grenni P., Gamboni M. (2017). Ecosystem Services Provided by Soil Microorganisms. Soil Biological Communities and Ecosystem Resilience.

[31] Rafiq M.K., Bai Y., Aziz R., Rafiq M.T., Mašek O., Bachmann R.T., Joseph S., Shahbaz M., Qayyum A., Shang Z. (2020). Biochar Amendment Improves Alpine Meadows Growth and Soil Health in Tibetan Plateau over a Three Year Period. Sci. Total Environ..

[32] Canarini A., Carrillo Y., Mariotte P., Ingram L., Dijkstra F.A. (2016). Soil Microbial Community Resistance to Drought and Links to C Stabilization in an Australian Grassland. Soil Biol. Biochem..

[33] Mureva A., Ward D. (2017). Soil Microbial Biomass and Functional Diversity in Shrub-Encroached Grasslands along a Precipitation Gradient. Pedobiologia.

[34] Li C., Fultz L.M., Moore-Kucera J., Acosta-Martínez V., Kakarla M., Weindorf D.C. (2018). Soil Microbial Community Restoration in Conservation Reserve Program Semi-Arid Grasslands. Soil Biol. Biochem..

[35] van Eekeren N., de Boer H., Hanegraaf M., Bokhorst J., Nierop D., Bloem J., Schouten T., de Goede R., Brussaard L. (2010). Ecosystem Services in Grassland Associated with Biotic and Abiotic Soil Parameters. Soil Biol. Biochem..

[36] Pullaiah T., Bahadur B., Krishnamurthy K.V., Bahadur B., Venkat Rajam M., Sahijram L., Krishnamurthy K.V. (2015). Plant Biodiversity. Plant Biology and Biotechnology: Volume I: Plant Diversity, Organization, Function and Improvement.

[37] Chang J., Ciais P., Herrero M., Havlik P., Campioli M., Zhang X., Bai Y., Viovy N., Joiner J., Wang X. (2016). Combining Livestock Production Information in a Process-Based Vegetation Model to Reconstruct the History of Grassland Management. Biogeosciences.

[38] Vitousek P.M. (2015). Grassland Ecology: Complexity of Nutrient Constraints. Nat. Plants.

[39] Nkuekam G.K., Cowan D.A., Valverde A. (2018). Arable Agriculture Changes Soil Microbial Communities in the South African Grassland Biome. S. Afr. J. Sci..

[40] Denef K., Roobroeck D., Manimel Wadu M.C.W., Lootens P., Boeckx P. (2009). Microbial Community Composition and Rhizodeposit-Carbon Assimilation in Differently Managed Temperate Grassland Soils. Soil Biol. Biochem..

[41] Millard P., Singh B.K. (2010). Does Grassland Vegetation Drive Soil Microbial Diversity?. Nutr. Cycl. Agroecosyst..

[42] Arcand M.M., Helgason B.L., Lemke R.L. (2016). Microbial Crop Residue Decomposition Dynamics in Organic and Conventionally Managed Soils. Appl. Soil Ecol..

[43] Xu W., Ge Z., Poudel D.R. (2015). Application and Optimization of Biolog EcoPlates in Functional Diversity Studies of Soil Microbial Communities. MATEC Web Conf..

[44] Zhen T., Fan W., Wang H., Cao X., Xu X. (2020). Monitoring Soil Microorganisms with Community-Level Physiological Profiles Using Biolog EcoPlatesTM in Chaohu Lakeside Wetland, East China. Eurasian Soil Sci..

[45] Wolinska A., Frąc M., Oszust K., Szafranek-Nakonieczna A., Zielenkiewicz U., Stępniewska Z. (2017). Microbial Biodiversity of Meadows under Different Modes of Land Use: Catabolic and Genetic Fingerprinting. World J. Microbiol. Biotechnol..

[46] https://www.biolog.com/support/bibliography/.

[47] Chabrerie O., Laval K., Puget P., Desaire S., Alard D. (2003). Relationship between Plant and Soil Microbial Communities along a Successional Gradient in a Chalk Grassland in North-Western France. Appl. Soil Ecol..

[48] Dengler J., Richardson D., Castree N., Goodchild M.F., Kobayashi A., Liu W., Marston R.A. (2017). Phytosociology. The International Encyclopedia of Geography.

[49] Tilman D., Downing J.A. (1994). Biodiversity and Stability in Grasslands. Nature.

[50] Vaida I., Păcurar F., Rotar I., Tomoș L., Stoian V. (2021). Changes in Diversity Due to Long-Term Management in a High Natural Value Grassland. Plants.

[51] Chytrý M., Hejcman M., Hennekens S.M., Schellberg J. (2009). Changes in Vegetation Types and Ellenberg Indicator Values after 65 Years of Fertilizer Application in the Rengen Grassland Experiment, Germany. Appl. Veg. Sci..

[52] Floch C., Chevremont A.-C., Joanico K. (2011). Indicators of Pesticide Contamination: Soil Enzyme Compared to Functional Diversity of Bacterial Communities via Biolog^®^ Ecoplates. Eur. J. Soil Biol..

[53] Rutgers M., Wouterse M., Drost S.M. (2016). Monitoring Soil Bacteria with Community-Level Physiological Profiles Using BiologTM ECO-Plates in the Netherlands and Europe. Appl. Soil Ecol..

[54] Schloter M., Nannipieri P., Sørensen S.J., van Elsas J.D. (2018). Microbial Indicators for Soil Quality. Biol. Fertil. Soils.

[55] Stone D., Ritz K., Griffiths B.G. (2016). Selection of Biological Indicators Appropriate for European Soil Monitoring. Appl. Soil Ecol..

[56] Buttigieg P.L., Ramette A. (2014). A guide to statistical analysis in microbial ecology: A community-focused, living review of multivariate data analyses. FEMS Microbiol. Ecol..

[57] Corcoz L., Păcurar F., Vaida I., Pleșa A., Moldovan C., Stoian V., Vidican R. (2022). Deciphering the Colonization Strategies in Roots of Long-Term Fertilized Festuca rubra. Agronomy.

[58] Weber K.P., Legge R.L. (2009). One-dimensional metric for tracking bacterial community divergence using sole carbon source utilization patterns. J. Microbiol. Methods.

[59] Garland J.L. (1997). Analysis and interpretation of community-level physiological profiles in microbial ecology. FEMS Microbiol. Ecol..

[60] RStudio Team (2019). RStudio: Integrated Development Environment for R.

[61] RCore Team (2021). R: A Language and Environment for Statistical Computing.

[62] Oksanen J., Blanchet F.G., Friendly M., Kindt R., Legrende P., McGlinn D., Wagner H. Vegan: Community Ecology Package, R Package Version 2.5-2; 2019. https://CRAN.R-project.org/package=vegan.

[63] De Mendiburu F. (2019). Agricolae: Statistical Procedures for Agricultural Research. https://CRAN.R-project.org/package=agricolae.

