# Supplementary material for: Exploration of Soil Functional Microbiomes—A Concept Proposal for Long-Term Fertilized Grasslands
Source: Plants (Basel). 2022 May 5;11(9):1253. doi: 10.3390/plants11091253 (PMC9102102; doi:10.3390/plants11091253)
Supplement: Supplementary file 1 [file plants-11-01253-s001.zip › Supp data.pdf]

Supplementary Data

Full description of the DEMSA model

The DEMSA model is built as an excel application with 3 worksheets. Working flux is based on the interconnectivity between the 3 worksheets, being necessary just one single introduction of raw data obtained by the reading of Biolog Ecoplates. For a dynamic presentation of the model, all the definitions are presented when they appear first time in the flux, located by the lines in the excel document. The entire document is designed for the comparison of 5 functional microbiomes shaped by treatments in 4 replications each, according to the research protocol from the article.

First worksheet (Database) – represent the place where are inserted the raw data (L1-L22), on their basis being computed the means for each variant and the further development of matrices related to complementary segments of ecological niche. Each substrate is considered as a **functional group**, and all the substrates that belong to the same chemical class form a **functional guild**. The native **ecological niche** represents the average pattern of microbial functional community, computed by the average of each functional group (L33). The sum of all those computed groups represents the average activity of native microbiome (L33/AI33). The only substrate that is excluded from all computations is water, because it presents only the basal respiration and thus it will serve as a primary comparison between treatments and is eliminated from the rest of the model.

Concepts and definitions

Index	Definition	Location in excel
• g_x	*Average of each functional group from control (except water)*	L33
• $\sum_x$	*Sum of all average functional groups – correspond to the average of the entire activity*	L33/AI33
• g_x%	*The % of each functional group in the average activity ($\sum_x$)*	L34

Raw data from control are further assembled for the defining of the limits, minimum and maximum, of ecological niche. In this context it can be determined the theoretical functional microbial pattern, both the minimum and maximum possibilities. The percent of average functional groups are used as a computation base along with the minimum and maximum sums of activities recorded from Ecoplates readings. The limits of native ecological niche (control variant) are defined by the theoretical minim and maximum values, with its average value used as the middle point of this niche.

Concepts and definitions

Index		Definition	Location in excel
•	$\sum_{min}$	*The minimum sum of functional groups registered in the control variants*	L35/AI35
•	g_{min}	*The calculated value of each group based on average percent (g_x%) related to the minimum sum ($\sum_{min}$)*	L35
•	$\sum_{MAX}$	*The maximum sum of functional groups registered in the control variants*	L37/AI37
•	g_{MAX}	*The calculated value of each group based on average percent (g_x%) related to the maximum sum ($\sum_{MAX}$)*	L37

Based on punctual comparison between each functional group in treated variants and average values, 2 transition matrices are constructed as a base for further computations. All the functional groups that present an equal or higher activity than the average of control is used in the construction of the "Base INCREASE matrix". As an opposite, all functional groups with lower activities than the average of control serve as values in the "Base DECREASE matrix". The future development of the model uses the "Base INCREASE matrix" to compute an INTENSIFICATION matrix" and an "EXPANSION matrix". Similar, the "Base DECREASE matrix" is used to compute the "NARROWING matrix" and the "CONTRACTION matrix". For the comparison of sharing patterns between treated microbial communities and untreated control, a "SHARING matrix" is computed in order to provide the image of global deviation compared to native community.

Concepts and definitions

Index		Definition	Location in excel
•	Base INCREASE matrix	*A matrix with all the values of functional groups equal or higher than the average of control*	L56-L74
•	Ag	*The value of each functional group that shows an increase compared to the average one (g_x)*	L56-L74
•	gI	*The calculated value for each Ag, that is in the interval g_x to g_{MAX}; if the value is higher than g_{MAX} it will be replaced by the value resulted from the difference between the 2 limits ($g_{MAX} – g_x$)*	L98-L116
•	INTENSIFICATION matrix	*A matrix of gI values which present the pattern of increases in the activity of functional groups compared to the average one, but within the limits of ecological niche*	L98-L116
•	gE	*The calculated value for each Ag that exceeds g_{MAX} based on the difference between them (Ag – g_{MAX})*	L77-L96
•	EXPANSION matrix	*A matrix of values which present the pattern of increases in the activity of functional groups, compared to the maximum value of potential activity (g_{MAX}), outside the higher limit of ecological niche*	L77-L96
•	Base DECREASE matrix	*A matrix with all the values of functional groups lower than the average of control*	L119-L137

• Dg	*The value of each functional group that shows a decrease compared to average value (g_x)*	L119-L137
• gN	*The calculated value for each Dg, that is in the interval g_x to g_{min}; if the value is lower than g_{min} it will be replaced by the value resulted from the difference between the 2 limits ($g_x - g_{min}$)*	L160-L178
• NARROWING matrix	*A matrix of gN values which present the pattern of decreases in the activity functional groups, compared to the average one, but within the limits of ecological niche*	L160-L178
• gC	*The calculated value for each Dg that are bellow g_{min} based on the difference between them (g_{min} – Dg)*	L140-L158
• CONTRACTION matrix	*A matrix of values which present the pattern of decreases in the activity of functional groups, compared to the minimum value of potential activity (g_{min}), outside the lower limit of ecological niche*	L140-L158
• SHARING matrix	*A matrix of values which present the pattern of sharing in the activity of functional groups, compared to the average value (g_x)*	L180-L199

The second worksheet (Community FR) – is used for the development of a set of indices that describe the magnitude of alteration suffered by microbial functional community due to the application of treatments. This worksheet provides another 2 synthetic indices. The first one – WCfr (*Within Community functional resemblance*) – shows the overlap of microbiome activity identified in the replications within the same treatment. This index scores the overlap by comparing each 2 replications. It is useful for the description of the stability of functional microbiome due to a treatment. Another use of the index is to present the potential divergence inside a community defined by the same treatment, and thus to show its perturbation potential. The second index – BCfr (*Between Community functional resemblance*) – shows the overlap between communities defined by different treatments. It is analyzed and interpreted in 2 forms. The first form is a General index – when each replication of one variant is compared to all of the replications from other variants. The second form is a Specific (one *x* one) index – based on the comparison of each 2 variants in order to identify the reduced functional resemblance between them. This form of BCfr is useful in the identification of fluctuations and successions in functional microbiome induced by treatments.

Concepts and definitions

Index	Definition	Location in excel
• **Functional alteration (Fa %)**	*The % of increases (+) of decreases (–) in microbial communities compared to the average control community*	AK3-AK18
• **Within Community functional resemblance (WCfr %)**	*An index of functional resemblance within the functional microbial communities that belong to the same treatment*	L24-L40

• **Between Community functional resemblance (BCfr %)**	*An index of functional resemblance between functional microbiomes that belong to different treatments*	L45-L62

The third worksheet (Percent Conversion) – is used for the identification of Dominance-Codominace patterns in functional microbiomes. All the data belonging to each of the 4 previous computed matrices: Intensification (L2-17), Expansion (L18-33), Narrowing (L34-49) and Contraction (L50-65) are converted to percent (%), related to the sum of activities for each treatment inside each matrix (AH column). This process result in a large matrix of percent located within the lines L67-L131. Based on their shares inside community, the Dominant-Codominant functional guilds and groups can be identified. The concept is based on phytosociology (the selection of dominant-codominant functional guilds) and will present the Guild association within a community; and on agronomical evaluation of grasslands (the selection of dominant-codominant functional groups) and will present the community assemblage. From an efficiency perspective, the selection of dominant-codominant guilds is located within the lines of raw data (L2-L55) with the selection of first 2 highest values and the conversion of guilds values in percent. The selection of dominant-codominant functional groups is located within the lines of converted data (L67-L131) with the extraction of the highest 2 values and the automatic fill of dominant cell in blue and the codominant one in green. The averages of each treatment inside matrices are computed for all of the values in the worksheet and located in the lines L133-149 for raw data and within lines L152-L168 for the percent converted data.

Concepts and definitions

Index	Definition	Location in excel
• **Guild association**	*Present the first 2 functional guilds based on their participation (%) within community*	Raw data L2-L65/AJ-BA Average data L133-149/AJ-AZ
• **Community assemblage**	*Present the first 2 functional groups based on their participation (%) within community*	Raw data L67-L131 Average data L152-168/AJ-AN

The second use of this worksheet is to determine the specific indices of Intensification, Expansion, Narrowwing and Contraction in the microbiome, due to the applied treatments.

This set of indices is designed to show the % of microbial activity within community associated to each of the complementary segments in ecological niche. The location of calculated indices is within lines L2-L65, on the column BE.
